# Reclaiming Indigenous systems of healing: experiences of disabled Māori of Māori-centric health service responses in Aotearoa New Zealand during the COVID-19 pandemic

**DOI:** 10.1186/s12913-024-12012-w

**Published:** 2025-01-06

**Authors:** Michael Roguski, Tara N. Officer, Gretchen Good, Karen McBride-Henry

**Affiliations:** 1 Kaitiaki Research and Evaluation, Wellington, New Zealand; 2 School of Nursing, Midwifery, and Health Practice, Wellington Faculty of Health, Victoria University of Wellington, Wellington, New Zealand; 3 School of Health Sciences, Massey University, Palmerston North, New Zealand

**Keywords:** Indigenous, Disabled, Healthcare services, Empowerment methodology, Kaupapa Māori, Māori, Holistic health services, COVID-19

## Abstract

**Background:**

The impact of the pandemic on Indigenous and disabled people's access to healthcare has resulted in significant disruptions and has exacerbated longstanding inequitable healthcare service delivery. Research within Aotearoa New Zealand has demonstrated that there has been success in the provision of healthcare by Māori for their community; however, the experiences of tāngata whaikaha Māori, disabled Māori, have yet to be considered by researchers.

**Methods:**

Underpinned by an empowerment theory and Kaupapa Māori methodology, this research explores the lived realities of tāngata whaikaha Māori or their primary caregivers. Twenty in-depth interviews gathered participants’ lived experiences, and a discursive lens was brought to the narratives of tāngata whaikaha Māori who have accessed, and received, culturally responsive healthcare services during the pandemic.

**Results:**

Positive experiences accessing primary and secondary services were associated with Māori-centred healthcare and seamless engagement with support services that were founded upon the active dismantling of structural inequities and the prioritisation of Māori cultural values in their care delivery, inclusive of tino rangatiratanga (sovereignty), and mātauranga Māori (Māori knowledge).

**Conclusions:**

This study provides a novel and solid foundation for comprehending how healthcare can be realigned to cater to the requirements of disabled Indigenous populations.

**Supplementary Information:**

The online version contains supplementary material available at 10.1186/s12913-024-12012-w.

## Introduction

Te Tiriti o Waitangi (The Treaty of Waitangi) has long been recognised as the founding document of Aotearoa New Zealand (Aotearoa). Signed in 1840, Te Tiriti afforded Māori, the Indigenous peoples of Aotearoa, tino rangatiratanga (self-determination, sovereignty, independence, autonomy) and a number of protections; including equal access to State-mandated services, and ōritetanga (equity) with British subjects [1]. Despite these protections, however, Māori have experienced a host of inequities, including healthcare access and associated outcomes. Notably, these outcomes have been traced to colonial *othering*, the subjugation of rights, and successive governments contravening Te Tiriti o Waitangi articles that secured the rights of Māori in partnership with the Crown [1].

With a particular focus on health, Māori have experienced long-term challenges accessing appropriate and culturally-responsive healthcare within Aotearoa [1]. Experts have linked monocultural service delivery, or a lack of culturally responsive healthcare service, to a raft of adverse health and social outcomes for Māori [1–5]. These include an inability to access relational healthcare and care that incorporates Indigenous healing modalities and recognition of peoples' spiritual needs [2–4, 6, 7]. As a consequence, Māori have commonly described healthcare services as hostile [8]; often resulting in disengagement from western (mainstream) services [1, 7]. As a result, Māori are frequently negatively portrayed in health-related statistics, whereby Māori either do not access or delay seeking healthcare, and when they do, experience substandard care that results in an increased burden of illness and disease [1, 2, 9].

### COVID-19

Aotearoa had one of the most stringent COVID-19 lockdown periods globally. International borders were closed to non-citizens, and strict isolation measures were in place for months for some parts of the country. Only limited research has explored the impact of healthcare access and disability support services for Indigenous people during the COVID-19 pandemic [4, 9–13]. However, Aotearoa-centred research completed by Dutta and colleagues (2022) reveals that the waiting times to see a health practitioner, the cost of healthcare, and new forms of healthcare delivery, such as telehealth, resulted in hardship for participants. Additionally, the inability to bring support people, or have one’s children, attend in-person health visits created inordinate barriers. The research team concluded that Māori, accessed “health at the margins” (p. 5) because of the structural barriers embedded in the health system; the pandemic having exacerbated previously identified inequities [4, 11].

During the pandemic, a shift in how Māori addressed and operationalised their own community was also recognised [14, 15]. Cram described how the concept of mahi aroha, loosely translated as ‘work driven by love’, drives Māori responses during crises such as the pandemic. Furthermore, research by Russell and colleagues (2023) highlights how Māori leaders and the health workforce mobilised to support their communities throughout Aotearoa. This mobilisation was achieved regardless of official government support or funding but because of a sense of responsibility and recognition of the hardship such crises created [14, 15]. Community leaders, across multiple spheres, were reported as successfully driving the mobilisation of Kaupapa Māori responses and services, which are guided by Māori cultural values and practices in addressing the holistic needs of whānau (extended family) and the wider community and involved activating various community networks.

### Tāngata whaikaha Māori

The success of Kaupapa Māori responses helps frame the experiences of the wider Māori community during the pandemic, but the experiences of tāngata whaikaha Māori, disabled Māori, have not yet been considered. Research over this period has emphasised the challenges for disabled populations more generally in accessing healthcare [16, 17]; this is especially true for marginalised populations groups [2, 10, 13, 18, 19]. Many of the challenges for disabled populations stemmed from a reduction or cessation of disability services that support disabled people to access healthcare, medication, and transport to healthcare providers. Therapies that helped health and general wellbeing were also negatively impacted during the pandemic [20–23]. For example, disability support workers did not support disabled people because of lockdown restrictions, misinterpretation of the restrictions, and a shortage of support workers [24]. Equally, accessing healthcare has been challenging for those with specific disabilities; for example, Deaf people struggled to lipread because of mask use [17, 25–28] and vision impaired or blind people struggled with the reconfiguration of physical spaces [16, 29, 30].

Attempts to resolve a lack of access through the use of initiatives such as telehealth further compounded problems for some disabled people if they lacked access to disability support, phones or the internet or were unable to use accessible technology [22, 23, 31]. Irrespective of funding issues, telehealth was inaccessible for Deaf people, those with vision impairments, and memory loss due to a lack of technical and supporting structures [16, 17, 32].

We know that disabled people access care at double the rate of their non-disabled counterparts [13, 33] and are at risk of even more significant disparities because of unequal access [34]. We also know that people with compounding vulnerability, such as disabled people and historically excluded communities, experience poorer health outcomes during the pandemic [18, 29, 35, 36]. The research team was interested in exploring the experiences of tāngata whaikaha Māori, representing approximately 32% of the Māori population [37], who accessed Kaupapa Māori health services during the pandemic and how this impacted their perceived health status. Therefore, this study aims to explore the lived realities of tāngata whaikaha Māori in accessing and receiving culturally responsive healthcare services during the pandemic.

## Theoretical framing

This study is framed in accordance with Kaupapa Māori Theory, and in alignment with a social model of disability and empowerment research informed our work. Kaupapa Māori Theory, aligned with Critical Theory, is inherently political, and according to Linda Smith, frames Indigenous research inquiry with a specific emphasis on “notions of critique, resistance, struggle and emancipation”(p. 185) [38]. We defer to a social model of disability to highlight the role of ableism and the structural imposition of disability, namely imposed isolation and exclusion from full societal participation [39, 40]. Empowerment research theory acknowledges the socio-political history of disabled people's marginalisation. It directly opposes deficit models by reframing inquiry from deficits to competence, illness to wellness, and weaknesses to strengths. Similar to the social model of disability, empowerment research contends models of individualised deficiency or blame in favour of an appreciation of environmental influences. Empowerment research explicitly positions participant voices as central, and they are regarded as valid and reliable [41, 42].

## Methodology

A Kaupapa Māori methodology underpinned this research. As a result, Māori principles and values embedded and informed each stage of this research process [43]. Accordingly, te ao Māori (a Māori worldview and cultural understandings) is located as central to the methodology, and this drives all aspects of the research from the gathering, analysis of findings, and the presentation of narratives.

Considering the research aim, a qualitative design was chosen. Qualitative methods embrace the purposeful telling of stories to understand human experience and describe the meaning that these hold for people and are in keeping with a Kaupapa Māori research methodology [43]. In-depth interviews were used to gather narratives, and a discursive lens was brought to the analysis.

Ethics approval for this study was granted by Te Herenga Waka—the Victoria University of Wellington Human Ethics Committee (#30121). Eligibility criteria included: identifying as tangata whaikaha Māori; being at least 18 years old or being the parent/grandparent of a tangata whaikaha Māori child; and having accessed healthcare during the COVID-19 pandemic. Recruitment was conducted by the Māori research team member, and lead author (MR), and in keeping with te ao Māori principles a purposive sampling approach was employed. Verbal and written consent was gained from participants before the beginning of in-depth interviews that ranged from 60–90 min. The interview guide, designed specifically for the Māori participants, focused on exploring participant experiences and how service change could improve healthcare and disability support access during/ after a pandemic (please see Supplementary File 1). A combination of both te reo Māori and English were used during the interviews (refer to Table 1 for glossary). To acknowledge their time, and for gifting their experiences, participants received a $50 koha (gift) at the interview's conclusion.

**Table 1:** Glossary

Te Reo Māori	English Translation
Hapū	Constellations of whānau; subtribe
Hinengaro	Mental and emotional wellbeing
Kaitiaki	Guardian
Kaitiakitanga	Stewardship
Kaupapa Māori methodology	Māori approach to research and research practices
Mana	Life force of a person, self-determination, power, sovereignty, independence, autonomy
Manaakitanga	Kindness, respect and generosity for others
Marae	Courtyard where Māori gather in front of a meeting house
Mātauranga Māori	Knowledge and wisdom arising from Māori world standpoint
Mokopuna	Grandchildren
Taha tinana	Physical body
Tane	Man
Tāngata whaikaha Māori	Disabled Māori people
Tangata whaikaha Māori	Disabled Māori person
Tāngata whaikaha tauiwi	Disabled non-Māori people
Tauiwi	Non-Māori
Te ao Māori	Māori worldview
Te reo Māori	Māori language
Tikanga	That which is right
Tino rangatiratanga	Self-determination, sovereignty, independence, autonomy
Tīpuna	Ancestors
Wairua	Spirit and spirituality
Wahine	Woman
Whānau	Wider or extended family
Whanaungatanga	Social connectedness
Whakawhanaungatanga	Establishing good relationships and hospitality

Definitions source: Moorfield [44], Manatū Taonga Ministry for Culture and Heritage [45], and Wilson et al. [46]

A total of (*n* = 20) tāngata whaikaha Māori participated in the interviews. Participants' ages ranged between 30 and 78 years. Most participants identified as female (*n* = 13), two as male (*n* = 6) and one as non-binary. Ten parents or grandparents were interviewed regarding 13 disabled children in their care; their children's ages ranged between six and 18 years. Children's disabilities included sensory, physical, and neurodivergence. The participants resided across rural and urban regions of Aotearoa.

Of the twenty participants, eight specifically discussed positive outcomes when they accessed healthcare, which is important when considering the structural and outcome-related negative statistics resulting from inherently racist health systems. Their experiences of accessing culturally responsive healthcare are the focus of this analysis.

### Data analysis

The interviews were audio-recorded, transcribed verbatim and subsequently returned to participants for any amendments, none took up this opportunity. Once interview transcripts were finalised a discursive analysis of the transcripts was conducted in keeping with the principles intrinsic to te ao Māori, such as tino rangatiratanga, kaitiakitanga, whanaungatanga, and manaakitanga. The development of the discursive themes was supported by the involvement of participants, with the presented findings reflective of their experiences as described in their words. At the conclusion of the analysis, results were presented to tāngata whaikaha Māori, and their feedback was used in the refinement of the final analysis. To support the research process, the non-Māori research team provided support to situate the analysis within national and international literature.

## Findings

### Context

Participants described mixed experiences accessing healthcare and receiving support during the pandemic. Within these varied experiences, the locus of care [47] was a primary determinant of whether tāngata whaikaha Māori experienced barriers to accessing healthcare and whether they received support.

Those who reported positive experiences were overwhelmingly engaged with either a Kaupapa Māori primary care provider or a mainstream provider, comprising tauiwi and Māori medical staff, who actively adhered to a holistic and culturally informed model of care. Notably, the latter mainstream providers employed a practice model founded on a dual commitment to te ao Māori (see *key tenets of te ao Māori* below) and a commitment to Te Tiriti o Waitangi; a commitment that challenges the pre-eminence of western biomedical individualism. Hereafter, Kaupapa Māori and mainstream providers committed to engaging and responding to Māori in accordance with te ao Māori are referred to as Māori-centred practices.

Significantly, tāngata whaikaha discussed healthcare and disability supports as seamlessly intertwined. Further, the likelihood of positive experiences was not linked to the severity of disability, namely whether an individual possessed a singular disability or fell into a categorisation of high and complex needs. Notably, all participants engaged in Māori-centred primary care asserted a belief that they would not have received such positive experiences throughout the COVID-19 pandemic if they had been engaged with western providers.

In contrast, similar to previously described accounts of tāngata whaikaha tauiwi, negative experiences were generally shared by those who accessed western primary medical care [16–18]. In addition, those with a singular non-immunocompromised impairment or disability, such as deafness or visual impairment, were more likely to report extensive access-related barriers. Significantly, participants reported that none of the western primary care practices provided supportive services or follow-up. As a consequence, compared to those engaged with Māori-centred practices, western affiliated participants reported heightened levels of isolation, discontinuation of care, and adverse effects such as decreased mobility, extreme isolation, and the development or aggravation of mental ill health.

The following analysis explores participants' perceptions of Māori-centred healthcare practices and why such practices resulted in positive experiences of accessing healthcare during COVID-19. Notably, participants commonly referenced past negative experiences with western health providers, and these are included to contextualise experiences with Māori-centred healthcare and support practices.

### Māori-centred healthcare and support

Two primary attributional themes were identified. Positive experiences were associated with Māori-centred healthcare and seamless engagement with support services because these practices: 1. reflected a commitment to key values and cultural understandings integral to te ao Māori; and 2. the active embedding of ōritetanga, the practice of equity, in the development and function of their service delivery.

#### Key tenets of te ao Māori

Māori-centred health care, and especially Kaupapa Māori practices, were regarded as exercising tino rangatiratanga in responding to the needs of tāngata whaikaha Māori, and Māori in general, by ensuring te ao Māori norms and practices were embedded as a central practice function. Such cultural adherence was described as uniquely positioning Māori-centred practices as upholding the individual's mana—a commitment that is in part aligned with western conceptualisations of person-centredness [48] but was framed as especially important as it stands in stark contrast to a transactional and "dehumanising" relationship that many participants had experienced with western services. Integral to these discussions was a sense of feeling "safe" and having experiences devoid of judgment.

*They [Māori-centred practices] also have a human quality about them and they're treating you like a person; they're not just treating you like a patient. (Tane, 32 years)*

*If I was to explain it to tauiwi [non-Māori]; in the mainstream system I feel like a number. Under a Māori-based system I feel like I'm a person. (Tane, 76 years)*

The upholding of the individual's mana was intricately linked to manaakitanga, the process of showing respect, generosity, and care for others. Manaakitanga was described as resulting in a sense of feeling welcomed and was commonly referenced as *"not feeling rushed"* when the individual met with their medical provider or the practice's nurses.

*It's manaakitanga and it's deep seated. As Māori, we live it and breathe it and, if the practice is truly Kaupapa Māori, you can see it in the way they treat their patients and the way the patients relate to them. (Wahine, 65 years)*

Finally, central to te ao Māori is a holistic appreciation of wellbeing that is intricately linked to a collective notion of self [49]. Within this context, Māori-centred practices exercised a holistic appreciation of self by intricately positioning the individual in relation to their whānau and hapū. Further, a holistic appreciation of wellbeing was linked to the increased likelihood of non-medical support being provided to the individual and whānau (discussed further below). Participants contrasted such commitment against western individualism and the often-singular focus on physical health in mainstream practices.

*The average mainstream provider or support service focuses on the person who's positive for COVID. They wouldn't necessarily be thinking about the needs of the other family members in the whare [house]. (Wahine, 65 years)*

#### Ōritetanga

Those accessing Māori-centred healthcare practices were universally aware of tāngata whaikaha experiencing disproportionately poor health outcomes [1] and participants shared their negative mainstream healthcare experiences to elucidate the importance of Māori-centred healthcare. Common to these descriptions were experiences of interpersonal and institutional racism whereby participants experienced inordinate delays, and the minimisation or disregard of symptoms. Aligned with such inequities, participants described the western health system within an embattled discourse; requiring the individual, and whānau, to consistently advocate to be heard.

*We have had to become self-advocates. Whether or not we have a positive healthcare experience is really dependent on how much we push to get what we need to make sure that we get well. (Tane, 46 years)*

Participants also discussed that inequitable health provision had resulted in their, or their whānau, past disengagement from healthcare practice and had contributed to a slew of negative outcomes.

*If you don't get a quality service, an effective service, or people are not nice, then you're just not going to go back. I mean, you know, it's like going to a shop to buy milk. If that person at the front desk is not nice to you, then you're not going to go back to that shop. (Tane, 66 years)*

In contrast, those accessing Māori-centred practices generally described a seamless provision of healthcare during the COVID-19 pandemic. The success of which was attributed to the intersection of cultural identity and practices, as underscored by the aforementioned te ao Māori tenets, and a principle of ōritetanga that acknowledges current and historical institutional barriers to Māori healthcare access while simultaneously working to ensure equitable access and healthcare delivery.

Participants often linked mana, manaakitanga, whānau, and ōritetanga as co-occurring functions of Māori-centred practice whereby person-centred care is intricately linked to the development of mechanisms, including advocacy, to minimise poor care provision for whānau. Consequently, participants often described seamless provision of disability support services, vaccination, primary, and secondary healthcare experiences.

#### Primary care

Multiple examples were provided of Māori-centred practice demonstrating a commitment to mana enhancing and equitable models of care. Within this context, primary care practices amended their mode of service delivery to accommodate the needs of tāngata whaikaha. Interactions, such as consultations being undertaken in the practice carpark and a significant shift to telehealth, greatly reduced the burden on the individual and whānau.

*They were very helpful. They actually, at one point, came out to me rather than me go into the clinic. They came out to the car to do tests and things like that. They were very helpful. (Tane, 76 years)*

Telehealth was especially appreciated amongst those with chronic conditions who viewed pre-COVID requirements for in-person consultation as an unnecessary formality. COVID-19 associated accommodations were viewed as a positive shift towards the empowerment of tāngata whaikaha and removed burden on the individual accessing care.

*It was just so much easier accessing services because of COVID. And being able to do things through phone calls and telehealth was just awesome… I'm a big fan of the whole telehealth and phone call system especially for those who are chronically ill. I mean, people who have longstanding chronic conditions know things like taking obs [observations] like pulse, blood pressure, neuros [neurological observations]. Like, "Is your strength on one side altered to the other". "Can you push against that?" You know, "On a scale of 1 to 10, how strong is that?" (Wahine, 45 years)*

Participants stressed that a significant component of their positive experiences with Māori-centred practices centred on holistic models of care, namely the provision of support external to conventional primary care, and that such holistic provision, commonly referred to as "wraparound support", had a significant impact on their wellbeing. Notably, support differed by practice and whether primary care practices had a social service arm attached to the practice or whether support occurred because of a preexisting relationship with a Māori social service organisation, with whom the individual's details were shared.

Similar to previous comments about the transactional nature of participants' experiences with mainstream services, participants, in part, traced their positive experiences to the emphasis of relationships underpinning their engagement with supportive services. The emphasis on relational aspects of care reinforced the person-centred nature of the relationship and minimised administrative requirements and interactions associated with mainstream service delivery that negatively impacted on the individual's likelihood of engaging with primary care.

*Under a Māori-based framework, when it fully goes that way, or as much as it can, I feel like I'm a person. Obviously, they have rules. Everyone has rules. But the hauora doesn't let the rules limit their services. To them, the rules are guidelines. But ultimately, each individual is an individual. And they will give you the best support they can based on what they know about you personally. (Tane, 32 years)*

*It wasn't just the support worker who knew me. The coordinator knew me, and the coordinator's boss knew me. And they would keep me in the loop, and they would say, "Hey, it says on your records that you haven't got your second vaccination". But it wasn't just text, it was a personal phone call or a visit. (Tane, 66 years)*

Throughout Level 3 and 4 lockdown periods (i.e., those times where the most stringent stay-in-place orders applied), support was provided via regular telephone or text communication to inquire about the individual's well-being. Additionally, updates pertaining to vaccination schedules and locations were also shared. Most common was the provision of food parcels, delivered on a regular basis to the individual's home. In other situations, assessments were conducted to determine what additional supports were required. As lockdown levels eased, the various support services would continue through home visits.

*There were support services coming out of every orifice. I mean, they kept turning up on our doorstep. "Is there anything that we can get you?" I developed a new faith in our local support services and I'm really grateful. (Wahine, 45 years)*

The following excerpt is taken from an interview with a grandmother who cares for her two teenage grandchildren, one of whom is autistic. The excerpt is significant as it details how holistic support was provided to the whānau as a whole and not only to the individual diagnosed with COVID-19; a provision that is in contrast to western individualism and acknowledges that one family member’s illness has an effect on the whānau system.

*I provide care to my two grandsons. One has severe autism. The one without autism was diagnosed with COVID. The day after he was diagnosed, the local Iwi social services contacted me, as my hauora had given them my details… The conversation that we’d had initially was just about my moko [grandchild] testing positive and what they could do to help and then they came round with a food parcel… It was like they thought of everything. They had things like washing powder, dishwashing liquid, toilet paper, and food. But they thought of all the other things that you potentially couldn't go out and get because, you know, you had to stay home. They would call me everyday, and it was during one of those calls they said, "Can we help you with anything else…” I was talking about my other one, my moko with autism, and she says, "Well, I've got a colleague who's looks after people with disabilities". And that's how she put me on to X. X rang that afternoon, and he would ring, and we discussed strategies of how to help my moko with autism during lockdown. (Wahine, 65 years)*

#### Secondary care

Prior to COVID-19 participants described a host of negative experiences with secondary care. These experiences demonstrated an acute awareness of structural inequities within hospital settings that required the reliance on whānau members, or those in their social network, to advocate for appropriate and equitable treatment - including barriers to regular and transparent communication with medical professionals. The pandemic was generally described as exacerbating structural inequities. Preventative mechanisms, such as whānau advocacy and intervention, ceased with the imposition of hospital policies that prevented whānau from visiting. As a consequence, whānau members' ability to monitor the individual's care, and advocate when necessary, was curtailed.

Further, participants described western medicine's individualistic focus as inadvertently excluding social support structures, such as whānau, from medical consideration. The pandemic, and associated infection control measures, exacerbated whānau exclusionary practices and most participants shared that they spent considerable time during their hospitalisation worrying about the wellbeing of their whānau, both in terms of their health and whether their basic needs, such as food, were addressed. Such concern can be especially appreciated because many participants described that their whānau members lived with chronic conditions, which negatively positioned them should they contract COVID-19, but also resulted in compromised ability to access resources, such as food. Within this context, two secondary care-related mechanisms were identified as effectively minimising the impact of structural inequities. Notably, complementing a holistic appreciation of self, each successful account occurred in conjunction with the provision of support external to conventional understandings of medical care.

First, those aligned with Māori-centred practices were more likely to describe seamlessly accessing secondary care because their general practitioner (GP) removed access-related barriers. As a means of elucidation, the following excerpt describes the role of a GP who contacted the hospital emergency department (ED) to forewarn that their chronically ill patient had tested positive for COVID-19 and was waiting to be seen; an intervention that circumnavigated a projected five-hour wait.

*I rang the Healthline [national telephone-based health advice line] and I told them what was happening for me, and they actually got in contact with my GP and my GP rang me to say, "Where are you?" And I said, "I'm sitting in the ED waiting room". And then it was not long after that conversation somebody came and grabbed me. It was a five-hour wait but I was seen really quickly because my doctor had advocated for me. He phoned them and told them that I had pre-existing health issues and that I needed support. (Tane, 46 years)*

Second, prior to COVID-19, one hospital was noted as having implemented an equity-centred strategy to mitigate structural secondary healthcare-related inequities. The strategy centred on a collaboration with a local Māori social service organisation who provided in-hospital cultural support and patient advocacy, a strategy that was primarily geared towards Māori but open to non-Māori who requested additional support. Those providing support were formally referred to as kaitiaki, a concept that embodies guardianship or protection.

Of those hospitalised, only one participant was hospitalised within an equity-centric setting. Their account is informative as it signifies the ability of secondary healthcare settings to ensure appropriate treatment, communication, and holistic appreciation of self, when framed in accordance with equity considerations. This patient described what he found to be "an exemplary experience". The following excerpt describes the role of the kaitiaki in ensuring the participant remained fully informed about their health, whilst simultaneously ensuring the needs of their whānau were addressed. Notably, within the spirit of ōritetanga, kaitiaki served an invaluable function of reducing the risk of inequitable medical care and decreasing the possibility of supportive services not being offered.

*The kaitiaki aren't a social service. Rather they check up to see if you are receiving a quality service. That people are responding to your needs and you are getting your medication. They acknowledge that our GPs are part of our support whilst we are in the hospital as well. So, they made sure that my GP was checking in with me. My GP rang me on a daily basis to check in with me but one day he didn't, and they rang him, and he rang me straight back. They also checked in with my wife and my baby daily. We have friends here [in the region], but my wife isn't someone who would ask our friends for help. So, the kaitiaki arranged a massive food delivery to my wife and my son. It took a weight off of me knowing they were being looked after… Because of the kaitiaki, I felt through the whole experience of that, I never really had any pushing to do it… everything was handed to me on a platter, which really, really nice. (Tane, 46 years)*

### The inability to provide fully holistic care

Despite the success associated with the above interventions, the ability of Māori-centred primary care practices to ensure the holistic care of their tāngata whaikaha Māori was prevented by a disconnect between government contracted support services and the primary care practice. All participants living with mobility and sight-related disabilities shared that their home care support either abruptly ended or was severely curtailed with the imposition of the Level 3 and 4 lockdowns; resulting in distress, the inability to access food, and preventing many from being able to bathe and engage in personal hygiene. In the short-term participants described heightened levels of isolation and loneliness, this was especially noted for those living alone.

*And because I lived alone at the time, I was pretty much stuffed. My house was not looking good. I couldn't go do shopping. I was pretty much stuck at home and isolated, and I couldn't get much support. And it didn't change much throughout Level 3 as well. (Tane, 32 years)*

In the short-term, those with severe mobility impairments described a lack of personal care support that resulted in compromised health and, in some cases, hospitalisation arising from hygiene-related infections. In the medium term, this cohort described that a failure to provide in-home care, or access physiotherapy, resulted in decreased function, which they do not believe is recoverable.

*I need home care to help with putting things away and mopping the floors and all that. And they basically just said at the time, "We can't do that. We're not legally allowed to". And I also need personal care. And they're only allowed to do like very basic personal care. And even then, it was very limited because the support workers they had on staff, they had to limit as well. So sometimes you couldn't even get your personal care done so. I'm pretty mobile but still need help with certain things like drying my legs because I can't move my legs. And if I don't get them dried properly, they are prone to infections. And that's what happened… in the first lockdown I got an infection and ended up in hospital. That was scary. (Tane, 32 years)*

### Future considerations

Participants were acutely aware of the high variability in care quality across the nation and stressed the importance of standardising positive practices to ensure the removal of healthcare-related barriers and embedding universally accessible healthcare.

*I would be one of many that have received an excellent quality service. I wish everybody could experience it and that it was duplicated throughout the country. That way everyone is supported. (Tane, 46 years)*

Specifically, extrapolating from their experiences, participants stressed the need for primary care practices to develop strategies to ensure that disabled people are appropriately supported in emergency and pandemic situations. Two key strategic components were identified.

#### Continuity of care

The discontinuation of home-based care over Level 3 and 4 lockdowns resulted in participants being placed in severely compromised situations. At the least, care discontinuation resulted in isolation and the inability to conduct daily activities such as shopping for food and medicines. At the more extreme, care discontinuation left individuals unable to attend to personal hygiene which, in some situations, resulted in hospitalisation. Within this context, participants stressed the need for primary care to include homebased care. Notably, for tāngata whaikaha Māori the current separation of primary care and disability support services was seen as nonsensical as the two are interdependent and a failure to meet the support needs of individuals severely compromised their overall health.

*Wraparound support is really important. In a time like COVID, I think it should be common sense that people who are more vulnerable need different support because it's a unique time, right? (Tane, 32 years)*

#### Develop a disabled person support strategy

Participants recommended primary care practices develop systems to identify disabled people and then provide wraparound support during disaster and pandemic situations. Such pre-emptive communication strategies are essential because of inherent vulnerabilities and the fact that it cannot be assumed that tāngata whaikaha Māori reside with others who can provide necessary care or assistance with accessing medication, food or other resources.

*Clinics need to set up support systems where they know who their at-risk people would be. So at times like a pandemic they have a team that's able to contact them to see if they are okay and if they need anything. It's not complicated at all. I mean, how hard could it be for a couple of volunteers to just sit there and just call people. And just go and visit. If they're on their own, if they don't have friends, or if they don't have family, the isolation is horrendous. (Wahine, 65 years)*

## Discussion

Māori and Indigenous people worldwide have been consistently negatively positioned in terms of a raft of social determinants of health, socio-economic status, and overrepresentation in rates of incarceration. Most of the literature aims to understand the positioning of Indigenous populations in terms of social outcomes. This study stands apart by highlighting the various benefits linked to Māori wellbeing, tino rangatiratanga, and mātauranga Māori, which led to tāngata whaikaha Māori having in some cases effortless access to healthcare services across public, primary, and tertiary services in Aotearoa.

Research has highlighted that those with compounding vulnerabilities have been most affected by a lack of disability support and healthcare access during the pandemic [10, 18, 50]. For example, those from ethnic minority groups who are disabled were most at risk of inequitable access to healthcare [10, 13, 18, 50, 51]. The compounding impact of inequalities in healthcare access has also directly impacted Indigenous populations globally [4, 10–12]. However, our study highlights the counter positive experiences and lived realities of tāngata whaikaha Māori in accessing and receiving culturally responsive healthcare and disability services during the COVID-19 pandemic.

The study's findings show that those who engaged in Māori-centred or Kaupapa Māori practices perceived reduced obstacles in accessing primary care. Participants reported receiving holistic support beyond western medicine's typical focus on the physical, which was beneficial for them. Such support involves more than just integrating service provision and encompasses a move towards comprehensive healthcare delivery. Furthermore, these findings aligns with previous research on holistic Kaupapa Māori views on health, which encompass spiritual, familial, cultural, mental, and physical aspects as integral components of overall wellbeing [48, 49]. Significantly, these components frame access to health and disability support services as a culturally informed model of care; a model that stands in stark contrast to the experiences of those who attended western-derived healthcare services [1, 8].

The findings of this study emphasise the importance of integrating Māori cultural understandings and systems of healing in western healthcare provision. This is especially true in relation to the centrality of a holistic appreciation of health and highlights the need to reject reductionist conceptualisations, such as primary, secondary, and tertiary healthcare (see for example, Ewert and Evers [52], Pérez-Cuevas, Guanais [53]). Indeed, research has demonstrated that, due to its complexity, Indigenous populations often have a limited understanding of the healthcare system [52–54]. However, our study’s analysis has brought to light a new model of care that prioritises Māori-centred practices. The participants who accessed Māori-centric healthcare services found them to be highly beneficial and reported a high level of engagement. They acknowledged the staff's unwavering commitment towards removing systemic barriers that hinder and restrict equitable access to healthcare.

Participants described how staff within these organisations recognise the existence of inequities in healthcare that stem from institutional racism, and their goals were to act to remove these overlooked inequities. For example, access to healthcare support was holistic and considered the wider whānau needs. Interactions with healthcare professionals were based on cultural principles of manaakitanga (generosity, respect, and care) and mana-enhancing practices. Also, participants had immediate access to the level and type of healthcare they required. Additionally, home-based disability services were integral to healthcare services reducing the fragmentation and need to access different providers for care needs. Arguably, Māori-centred approaches to healthcare prioritise the removal of barriers and consider cultural needs, resulting in a substantial positive impact for tāngata whaikaha Māori when accessing this form of care.

Participants traced the success of their healthcare assess to specific key facets. These included Māori-centred healthcare, care embedded within a culturally responsive framework, whānau-centric healthcare and healthcare delivery that is founded upon principles of te ao Māori. A commitment to Māori-centred healthcare greatly minimised a raft of structural inequities. It is crucial that insights gained from this research on eliminating systemic inequities in healthcare are acted upon, and that Māori-centred models are implemented nationally.

### Implications for service delivery

It is essential to evaluate the disability and healthcare delivery systems in Aotearoa. Research within Aotearoa has repeatedly highlighted the inequitable access to healthcare for tāngata whaikaha Māori (see for example: Ingham, Jones [4], Espiner, Paine [8], Perry, Ingham [13], Reid, Paine [9], Waitangi Tribunal [1]). The healthcare system in Aotearoa, for myriad reasons, struggles to meet population healthcare needs; this has become particularly apparent because of the additional challenges wrought by the pandemic [1, 9, 13]. There are several important implications of this study, all of which focus on the emerging models of Māori-centric practices. By applying these models, we can improve the integration of healthcare services for Indigenous communities on a national level. This approach can help reduce healthcare disparities and ultimately lead to better health outcomes. The implementation of Indigenous-centric models in healthcare delivery worldwide can bring significant benefits by intentionally disrupting institutional racism and associated practices. These models are not only useful during normal periods but also particularly valuable during times of global health crises.

Focusing on a holistic understanding of health is crucial in providing Indigenous care [48, 55]. From a te ao Māori perspective, the wellbeing of the entire whānau is valued and prioritised, with emphasis on enabling all members to achieve optimal wellbeing. For the participants, holistic services involve a healthcare approach that is not focused solely on an individual but is whānau-centric and community focused, more in line with the principles of primary health care [12]. According to our participant group, healthcare provided by Māori-centric providers is authentically focused on whānau and their needs. This meant that care provision intentionally focused on providing healthcare for the entire whānau and not just the individual who contacted the healthcare service. Furthermore, having a single point of contact for all members of the whānau ensured that their individual needs were adequately addressed. This fostered a caring environment where the risk of people falling through the gaps was significantly reduced.

An essential part of providing holistic care involved merging healthcare and disability services. The participants who accessed Kaupapa Māori, and Māori-centric healthcare services described having benefited from the integration of these services. In Aotearoa, various non-government agencies provide different forms of disability support and primary care services through separate funding mechanisms. Disabled individuals accessing mainstream healthcare interact with several service providers, which can be challenging even in normal circumstances. However, the pandemic has highlighted the shortcomings of this approach, as it has led to failures in the provision of adequate disability and healthcare support services [10, 16–18]. Our group of participants emphasised that the inclusion of these services helped decrease their chances of feeling isolated and going hungry.

This research contributes to prior studies that showcase the effectiveness of Kaupapa Māori practices in meeting the needs of the Māori population [15]. Specifically, it delves into a previously unexamined Māori community to shed light on the experiences of tāngata whaikaha Māori who sought Kaupapa Māori healthcare during the pandemic. Additionally, the knowledge of, and willingness to respond from, a mātauranga Māori perspective allowed for the emergence of Māori-centric models of care; effectively demonstrating mātauranga Māori in practice. These unique models provided the freedom to flourish from within an Indigenous framework. However, the effort required by these healthcare communities was significant and often without funding, an issue that has also been described in Russell and colleagues' research [15].

### Limitations

This exploratory study centred on a select group of tāngata whaikaha Māori participants, in recognition of the community's distinctiveness and relatively small size. Although the number of participants was limited, the findings provide unique insights into accessing Indigenous-responsive healthcare. Specifically, the study examines the experience of accessing Māori-centred and Kaupapa Māori health service delivery. Considering the healthcare challenges faced by this community, it is crucial to comprehend how to deliver care in a culturally appropriate and whānau-centred manner that addresses their distinct requirements. This study sets a solid groundwork for future investigations on Indigenous disabled communities and offers valuable insights into the lived realities of accessing healthcare for this particular group.

### Conclusion

This study explored the experiences of accessing Māori-centred healthcare for tāngata whaikaha Māori, the Indigenous population of Aotearoa. The COVID-19 pandemic magnified the impact of healthcare access disparities within this population. Through the investigation, it was found that Kaupapa Māori services provide care based on cultural understandings. However, an important discovery was that non-Māori healthcare providers who implemented Māori-centric practices were also able to deliver improved healthcare; this was achieved by intentionally dismantling inequities and prioritising Māori cultural values in their delivery of care. This study provides a strong foundation for comprehending how healthcare can be realigned to cater to the requirements of disabled Indigenous populations.

## Supplementary Information

Supplementary Material 1.

## Data Availability

The de-identified datasets used and/or analysed during the current study available from the corresponding author on reasonable request.
