# Supplementary material for: Reclaiming Indigenous systems of healing: experiences of disabled Māori of Māori-centric health service responses in Aotearoa New Zealand during the COVID-19 pandemic
Source: BMC Health Serv Res. 2025 Jan 6;25:29. doi: 10.1186/s12913-024-12012-w (PMC11706141; doi:10.1186/s12913-024-12012-w)
Supplement: Supplementary file 1 — Supplementary Material 1. [file 12913_2024_12012_MOESM1_ESM.docx]

# Supplementary File 1

**Interview questions:**

1. Can you tell me about your experiences of accessing health care since the beginning of the pandemic?
   1. Prompt: explore the intersection between disability, gender, age, ethnicity, and family support structures.
2. Can you tell me about your experiences of accessing disability support services since the beginning of the pandemic?
   1. Prompt: explore the intersection between gender, age, ethnicity, and family support structures.
   2. Prompt: explore how the pandemic changed the experiences of accessing health and disability services.
3. Did you experience any barriers to accessing care or things that made it easier to access care? Can you tell me about these?
   1. Prompt: explore barriers to accessing care before and during the pandemic.
   2. Prompt: How have these experiences changed how you view health care access and the service provided?
4. How do you think these experiences have impacted your health during the pandemic?
   1. Prompt: Have you had any positive experiences that you can tell me about?
   2. Prompt: Have you had any negative experiences that you can tell me about?
5. How do you think that the pandemic has impacted your, or your child’s, long-term health?
   1. Prompt: explore the intersection between gender, age, ethnicity, and family support structures.
6. What would you like to see changed to help people access health care during a pandemic better?
   1. Prompt: explore any ideas related to healthcare and disability access.
7. Is there anything else that you would like to tell me about?
